# Speech-guided breathing retraining in asthma: a randomised controlled crossover trial in real-life outpatient settings

**DOI:** 10.1186/s13063-018-2727-z

**Published:** 2018-06-25

**Authors:** Dietrich von Bonin, Sabine D. Klein, Jana Würker, Eva Streit, Oliver Avianus, Christian Grah, Jörg Salomon, Ursula Wolf

**Affiliations:** 1 Institute of Complementary Medicine, University of Bern, Fabrikstrasse 8, 3012 Bern, Switzerland; 2 Klinik Arlesheim, Arlesheim, Switzerland; 3 Gemeinschaftskrankenhaus Havelhöhe, Clinic for Anthroposophical Medicine, Berlin, Germany; 4 LungenZentrum Salem-Spital, Bern, Switzerland; 5 Present address: Branch organisation of Swiss Arts Therapy associations, OdA ARTECURA, Utzigen, Switzerland; 6 Present address: Dresden, Germany

**Keywords:** Anthroposophic therapeutic speech, Asthma, Breathing, Lung function, Quality of life

## Abstract

**Background:**

Breathing retraining techniques have received increased attention in the management of asthma, because there is growing evidence of the usefulness of such methods in improving quality of life, reducing symptoms and reducing bronchodilator use. Our study investigated the effect of anthroposophic therapeutic speech (ATS), which uses sounds and syllabic rhythm to improve articulation, breathing and cardiorespiratory interaction, in patients with asthma in a real-life outpatient setting.

**Methods:**

In a randomised controlled crossover trial, patients with asthma in three centres in Switzerland and Germany were randomised to either receive 11 ATS sessions or to wait. Subsequently, patients changed either to wait or to receive ATS. Primary outcomes were changes from the beginning to the end of each phase in the Asthma Quality of Life Questionnaire (AQLQ) and spirometry parameters. Secondary outcomes were changes in inhaled glucocorticoids, the Asthma Control Test (ACT), peak flow and asthma exacerbations.

**Results:**

Altogether, 63 patients were randomised, of which 56 were enrolled and 49 completed the study. Statistically significant differences between the ATS groups and waiting control groups were found for the overall AQLQ score (*d* = 0.86, *p* = 0.001) and the domain scores for symptoms, activity limitation and emotional function as well as ACT score (*d* = 0.53, *p* = 0.048). No significant differences were observed in spirometry parameters, inhaled glucocorticoids, peak flow and days without asthma exacerbation per week. No serious adverse events occurred during ATS sessions.

**Conclusions:**

ATS significantly improves asthma control and quality of life in patients with asthma. Whether ATS may improve lung function remains to be shown.

**Trial registration:**

ClinicalTrials.gov NCT02501824. Retrospectively registered on 8 July 2015.

**Electronic supplementary material:**

The online version of this article (10.1186/s13063-018-2727-z) contains supplementary material, which is available to authorized users.

## Background

Breathing retraining techniques have received increased attention in the management of asthma, because there is growing evidence for the safety and usefulness of such methods. The Global Initiative for Asthma (GINA) reports evidence level B for the breathing techniques adjuvant to pharmacotherapy [1], and the British Guideline on the Management of Asthma similarly states a level of recommendation A for such techniques to improve quality of life, reduce symptoms and reduce bronchodilator use [2].

Physiotherapist-guided breathing programmes such as the Papworth method and the Buteyko method are the most systematically investigated and also yield the best evidence for effectiveness. The breathing programmes aim to reduce hyperventilation, strengthen nasal and diaphragmatic breathing, and reduce respiratory rate and minute volume [3]. The most consistent results favouring the intervention group have been found for quality of life (e.g. SF-36) and asthma related quality of life (e.g. Asthma Quality of Life Questionnaire, AQLQ) as well as in Asthma Control Test (ACT) scores [4]. So far, there is no convincing evidence for significant improvements in pulmonary function resulting from breathing interventions [2, 4]. However, an innovative recent study investigating physiotherapy-based breathing retraining demonstrated significant effects for end tidal CO_2_, breathing rate and predicted forced expiratory volume for the experimental group versus no additional treatment. The effects continued for 5 months, during which time patients practised the breathing behaviour learned in the training programme [5]. So far, there is insufficient evidence for the efficacy of breathing methods in the context of yoga, biofeedback and respiratory muscle training [2, 4, 6, 7].

Active breathing modulation techniques are frequently considered to be complementary medicine (CM) [6]. Even if such techniques are often not included in conventional asthma management plans, patients may take a different perspective, since the prevalence of CM in the treatment of asthma is 20–30% among adults and 50–60% for children, even if rigorous estimates are applied [8]. Breathing exercises have been found to be the most commonly used CM method [9].

Among holistic CM systems, anthroposophically extended medicine (AEM) is a well-integrated approach practised in both inpatient and outpatient settings by medical doctors and certified therapists. Currently, an AEM service is available in more than 50 countries [10]. AEM provides holistic health care, i.e. combining mainstream medicine with specific therapies. Prospective cohort studies have shown that it improves symptoms and the quality of life of patients with chronic diseases [11, 12]. Anthroposophic therapeutic speech (ATS) is one of the arts therapies, which aims at improving respiratory, vocal and articulatory functions by applying sounds and syllabic rhythm in combination with speaking poetry. Here, old forms of rhythmic verse, like Greek hexameter, have been shown to improve cardiorespiratory interaction and to evoke a calm and relaxed state of mind [13, 14]. ATS has been used in AEM for over 80 years and is provided by certified therapists in therapy centres and clinics for anthroposophic medicine [10]. The method has been used for many years as breathing retraining in the treatment of asthma.

Our study is the first to investigate the effects of ATS in patients with asthma in real-life outpatient settings. Based on our clinical experience, we assumed that 3 months of ATS including weekly training with a therapist and practising at home, would improve quality of life, asthma control and lung function. In particular, the following hypotheses were tested: (i) ATS improves asthma control and quality of life in patients with asthma, (ii) ATS improves relevant parameters of pulmonary function in patients with asthma and (iii) ATS reduces the application of reliever medications (used as needed) in patients with asthma.

## Methods

### Study design

The study was carried out as a randomised controlled multi-centre two-period crossover exploratory clinical trial conducted at three centres in Switzerland and Germany between October 2010 and August 2014. The trial was approved by the relevant ethical committees (Bern KEK 115/10, Basel EKBB 35/11 and Berlin KEK 115/10). It was registered post-interventions on 8 July 2015 on clinicaltrials.gov (NCT02501824), because when the trial was planned, the registration of non-pharmacological trials was not required in Switzerland. A Consolidated Standards of Reporting Trials (CONSORT) checklist is included in Additional file 1.

Participants were randomly assigned in a 1:1 allocation ratio to either receive 11 ATS sessions (one per week intended) or to wait (control). The patients were diagnosed and informed about the trial by the participating pneumologists. Patients interested in participating were referred to the study therapist, to receive and discuss an information sheet prior to providing written informed consent. The original study protocol included 11 conversation sessions with the same therapist as a control intervention. The protocol had to be modified after the third patient, as the patients were not complying with the 11 sessions of the control intervention, i.e. the conversations with the therapist. The conversation sessions were replaced by a waiting phase after approval from the ethical committee.

Blinding of the patients, therapists and physicians was not possible due to the nature of the intervention. Blinding of the statistician, who was part of the study team and had access to the primary data, was not attempted.

### Participants

Inclusion criteria were: 12 years or older, bronchial asthma for at least 1 year, inhaled a β_2_ agonist more than once per week and signed an informed consent form (or signed by a parent for a minor) prior to the beginning of the interventions. The diagnosis of asthma was made according to the current American Thoracic Society/European Respiratory Society recommendations [15]. Asthma severity had to be at least mild, persistent and not completely controlled. The diagnosis of asthma and exclusion of any other relevant airway disease was done by a respiratory physician.

Exclusion criteria were: severe systemic illness, coronary heart disease, severe chronic obstructive pulmonary disease, insufficient general condition to participate in an active therapy, pregnancy and current use of oral corticosteroids.

### Interventions

Interventions were administered in an outpatient centre (group practice: Therapeutikum Bern, Switzerland) and in the pneumology units of two clinics for mainstream medicine and AEM (Klinik Arlesheim, Switzerland and Gemeinschaftskrankenhaus Havelhöhe Berlin, Germany). Each participant was seen one-to-one by the same certified therapist for all training sessions and asked to practise for at least three times a week. ATS therapists have received full-time training in speech, drama and ATS for 4 years.

The training programme for each patient consisted of 10 frequently applied ATS exercises, which comprise spoken sound combinations of syllables (e.g. “Wwwwww-T” / “OM”) and rhyme (e.g. six lines of hexameter in German), adapted to the patient’s individual needs.^1^ The exercises have to be performed full-toned with the following aims: (a) extending and deepening respiration and making respiration rhythmical, (b) reducing hyperventilation, (c) improving sensations of stiffness and congestion in the chest and diaphragm and (d) opening airways by means of sound (e.g. syllables consisting of the vowel “A” together with consonants like “B”, “D” and “C”).

### Outcome parameters

The primary outcome measures were changes in AQLQ scores [16] and spirometry parameters: forced expiratory volume in 1 s (FEV1), forced vital capacity (FVC) and the ratio FEV1/FVC. The AQLQ was administered by the study therapists. Spirometry was measured in all centres by a pneumologist prior to the first phase, at crossover and after the second phase of the study.

Secondary outcomes were changes in inhaled glucocorticoids (in μg) per week, ACT score [17], peak flow and the number of days without asthma exacerbations per week. Participants kept daily diaries to record morning and evening peak expiratory flow measurements, medication use, exacerbations, worsening of condition, perceived pollen load, infections and holidays. The ACT questionnaire was filled out monthly.

To calculate changes in the outcome parameters, baseline values (i.e. measurements before a phase for primary outcomes or ACT and average values of the first 2 weeks of a phase for secondary outcomes) were subtracted from the values at the end of each phase. This was justified by the high correlation (>0.5) between baseline and outcome values [18].

### Sample size

No study on ATS in asthma patients was available as a basis to determine the sample size. We assumed a median effect size (*d* = 0.5), and set a power of 0.8 and α = 0.05. For paired data, *n* = 34 would be sufficient to detect a significant difference. Allowing for dropouts, we determined a sample size of 50 patients to finally reach *n* ≥ 34 for data analysis.

### Randomisation

The digits of π were used to generate the random allocation sequence, starting with the 151st digit (even digits stood for ATS and odd digits for waiting in the first phase). The random allocation sequence was generated by the statistician and managed by one therapist. Eligible patients were assigned to the two groups prior to the first information session with the therapist at which the informed consent had to be signed. The therapists enrolled the patients.

### Statistical methods

Analyses were performed using IBM SPSS Statistics for Windows, Version 23.0 (IBM Corp., Armonk, NY, USA). Normality of within-patient differences was verified using the Shapiro–Wilk test. *p* < 0.05 was considered statistically significant, and since this was an exploratory study, no adjustment for multiple comparisons was calculated. All tests were two-tailed. When within-patient differences were normally distributed (as for most primary endpoints), the dependent *t*-test was used to compare the two phases. For non-normally distributed endpoints, the Wilcoxon signed-rank test was applied. Missing data were not estimated.

Additionally, the same analysis was performed including only patients for which all data were available and whose medication intake was in accordance with the study protocol (a per protocol analysis).

## Results

### Patient characteristics

**Fig. 1:**
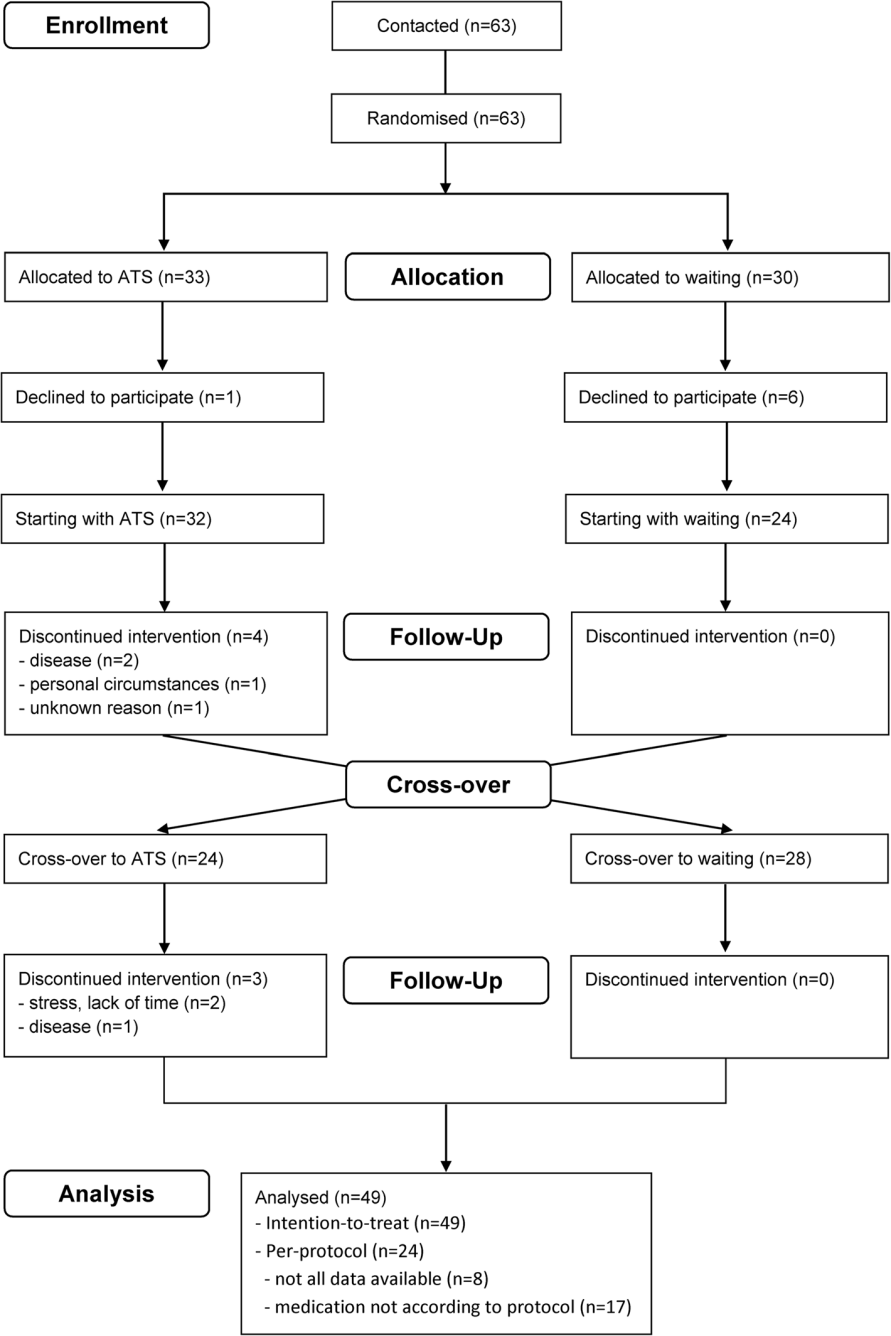
Flow of participants in the study. ATS anthroposophic therapeutic speech

Altogether, 63 patients with asthma were randomised, of which 56 were enrolled (28 in Arlesheim, 20 in Berlin and 8 in Bern) and 49 (87.5%) completed the study (Fig. 1). The baseline characteristics of the participants are shown in Table 1. The patients’ median FEV1/FVC was 69% and the ACT score 18 points. The median study duration per patient from the initial to the final medical examination was 182.5 days (interquartile range, IQR 167–219 days).

**Table 1:** Baseline characteristics of the trial participants

Characteristics	ATS/waiting (*n* = 32)	Waiting/ATS (*n* = 24)	Total (*n* = 56)
Age, years, median (IQR)	50 (42–62)	49 (40–60)	49 (42–61)
Female, no. (%)	21 (66)	17 (71)	38 (68)
Body mass index, kg/m^2^, median (IQR)	24 (22–26)	22 (20–25)	23 (21–25)
Asthma Control Test,^*^ median (IQR)	18 (14–22)	18 (15–21)	18 (15–22)
Asthma Quality of Life Questionnaire,^†^ median (IQR)	4.5 (3.6–5.3)	4.7 (3.8–5.2)	4.6 (3.7–5.2)
Lung function, median (IQR)			
FEV1, L	2.72 (2.02–3.38)	2.43 (2.02–3.05)	2.68 (2.03–3.20)
FEV1, %	88 (72–105)	85 (70–102)	85 (71–103)
FVC, L	3.54 (3.15–4.84)	3.43 (2.87–4.45)	3.52 (3.10–4.78)
FEV1/FVC, %	69 (63–77)	69 (62–75)	69 (62–76)

*ATS* Anthroposophic Therapeutic Speech, *FEV1* forced expiratory volume in 1 s, *FVC* forced expiratory vital capacity, *IQR* interquartile range

*Range 5–25, higher scores indicate less severe disease, score >19 indicates well-controlled asthma

^†^Range 1–7, higher scores indicate less impairment

### Effectiveness

Table 2 presents the treatment effects according to the intention-to-treat analysis. The AQLQ score increased by 0.63 points after ATS compared to 0.07 points after waiting, resulting in a 0.56-point difference between the two phases. Statistically significant differences between ATS and waiting were found for the overall score and the domain scores for symptoms, activity limitation and emotional function. No significant differences were observed in spirometry parameters, inhaled glucocorticoids, peak flow and days without asthma exacerbation per week. The ACT score rose by 1.57 points after ATS compared to −0.51 points after waiting, adding up to a statistically significant 2.09-point difference between the two phases.

**Table 2:** Treatment effects (intention-to-treat analysis)

Outcome	ATS, mean (SD) (*n* = 49)	Waiting, mean (SD) (n = 49)	Effect size, Cohen’s *d* (95% CI)	*p* value
Asthma Quality of Life Questionnaire				
Overall	0.63 (0.67)	0.07 (0.63)	0.86 (0.43 to 1.28)	0.001
Symptoms	0.60 (0.87)	0.03 (0.72)	0.71 (0.28 to 1.12)	0.006
Activity limitation	0.74 (0.76)	0.10 (0.76)	0.84 (0.41 to 1.25)	0.001
Emotional function	0.57 (0.85)	0.05 (0.91)	0.58 (0.17 to 0.99)	0.013
Environmental exposure	0.56 (1.03)	0.15 (0.84)	0.43 (0.02 to 0.84)	0.079
Lung function				
FEV1, L	− 0.01 (0.32)	0.05 (0.29)	− 0.18 (− 0.58 to 0.23)	0.471
FVC, L	0.02 (0.33)	0.03 (0.30)	− 0.06 (− 0.46 to 0.35)	0.805
FEV1/FVC, %^‡^	0.00 (0.09)	0.01 (0.10)	−0.01 (− 0.41 to 0.40)	0.385
Inhaled glucocorticoids, μg per week^†‡^	6.96 (189.08)	−8.35 (168.63)	0.09 (−0.31 to 0.48)	0.581
Asthma Control Test^‡^	1.57 (4.06)	−0.51 (3.78)	0.53 (0.12 to 0.94)	0.048
Peak flow (morning), L/min^‡^	8.27 (46.30)	5.42 (44.18)	0.06 (−0.35 to 0.47)	0.519
Peak flow (evening), L/min^‡^	9.24 (50.66)	9.71 (44.19)	−0.01 (− 0.42 to 0.40)	0.943
Days without asthma exacerbation per week^‡^	0.20 (1.27)	0.05 (0.98)	0.12 (−0.29 to 0.53)	0.758

Changes from the beginning to the end of the respective phase are presented. Dependent *t*-test was used if not stated otherwise

*ATS* anthroposophic therapeutic speech, *FEV1* forced expiratory volume in 1 s, *FVC* forced expiratory vital capacity, *SD* standard deviation

^‡^Wilcoxon signed-rank test

^†^In reliever medication

In addition, a per protocol analysis with 24 patients was performed, with similar results as in the intention-to-treat analysis. Statistically significant differences between the two phases were observed for the overall AQLQ score, activity limitation and emotional function (*p* = 0.015, *p* = 0.007 and *p* = 0.025, respectively). No significant changes were found in spirometry parameters (*p*(FEV1) = 0.631, *p*(FVC) = 0.961 and *p*(FEV1/FVC) = 0.889). The changes in the ACT score were no longer significant (*p* = 0.121).

**Fig. 2:**
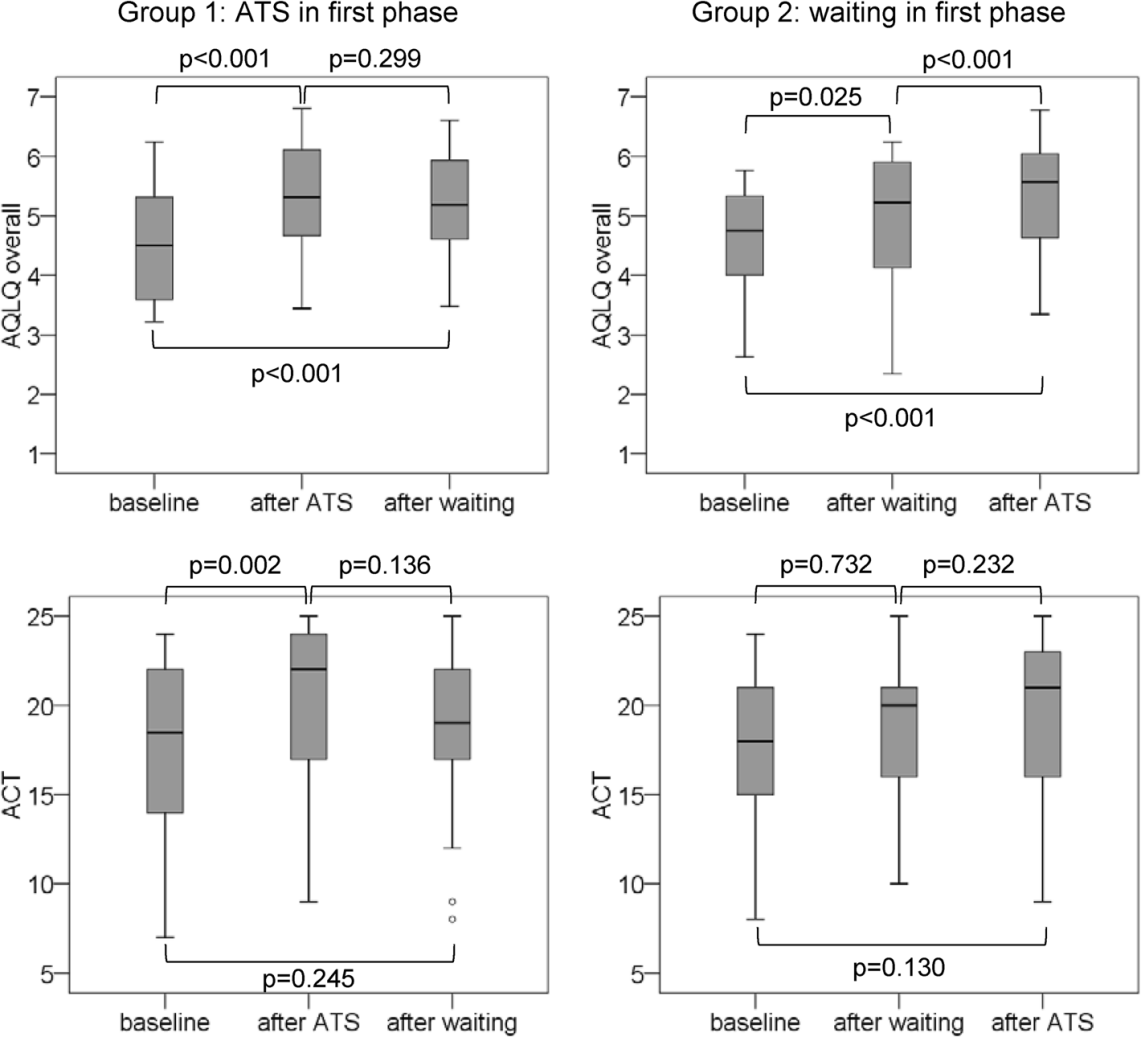
Box plots of overall AQLQ and ACT scores. Data at baseline, after waiting phase and after ATS, subdivided into the two groups are shown. Circles represent outliers. Spirometry parameters are not displayed, since no significant changes were observed. *ACT* Asthma Control Test, *AQLQ* Asthma Quality of Life Questionnaire, *ATS* anthroposophic therapeutic speech

There were indications for trend and/or carryover effects. In the group who waited during the first phase, a slight improvement in the overall AQLQ score and symptoms was noted during that phase (Fig. 2). In the other group, who first received ATS and then entered the waiting phase, the parameters (overall AQLQ score and all domain scores) did not return to the initial values.

### Safety

No serious adverse events occurred during ATS sessions or were reported by patients in the following sessions. However, 15 patients reported 22 minor adverse events to the therapists, which included difficulties in practising at home (9) (e.g. “I can’t practise when my partner is there”), worsening of condition during the waiting phase (5), high effort required to participate in the study (2), ATS phase too short to profit from the therapy (2), difficulty in connecting with the German language (1), hoarseness (1), headache (1), and cramping in the abdomen during one exercise (1). The therapists noted 20 minor adverse events in 14 patients. These included difficulties in performing the exercises (8), coughing (5) or a tickle in the throat (4), and hoarseness (3).

## Discussion

Of our three hypotheses, one was confirmed: ATS significantly improved asthma control and quality of life in patients with asthma. The AQLQ score rose by 0.56 points, 0.5 points being considered the minimal clinically important difference [19]. The ACT score rose by 2.09 points. We were not able to demonstrate that ATS improved the parameters of the pulmonary function tests or reduced the use of reliever medications in our study population of outpatients during the 11 weeks of treatment.

The baseline median ACT score of 18 points (<19) in our population as well as the median FEV1/FVC of 69% (<75%) indicated that asthma was not well controlled by our participants [20]. After ATS, the median ACT score rose to 20.09 points, corresponding to well-controlled asthma, even if the minimal clinically important difference is a change of 3 points [21]. This is consistent with the observations of patients, physicians and therapists, who expressed satisfaction with the clinical improvement after ATS.

All patients were regularly seen by a pneumologist and took conventional medication. However, we observed a low adherence to medication in some patients. These patients were excluded in a subsequent per protocol analysis, which gave similar results as the primary analysis. Low adherence has been observed in other investigations, too [22]. A population-based study showed that CM use was associated with poor asthma control that was independent of the level of intake of controller medication [9]. Taken together, these results may suggest that the patients in our study represent a special group of asthma patients who are suffering from difficult-to-control asthma and ready to learn active breathing retraining.

It is known that objective measures in the treatment of asthma only weakly to moderately correlate with how patients actually feel and are able to function in daily life. Thus, improvements in health-related quality of life may be rated more important than spirometry values, and they may be complementary outcomes [23].

The systematic review by Burgess et al. [6] identified only one trial in which pulmonary function changed during breathing retraining [5]. In this trial, the patients learned breathing retraining techniques in 12 individual sessions (three per week) and practised frequently at home (two or three times per day for 20 min) for 5 months. Other trials used fewer training sessions and a shorter training practice time of, e.g., 10 min/day. Those trials showed no improvement in pulmonary function. Responses in quality of life and asthma control parameters may, thus, be attained earlier than for pathophysiological outcomes.

Whether ATS improves lung function remains to be shown. ATS uses elements of language readily available to every speaking person and modifies the depth, rhythm and duration of exhalation by means of sound and syllable combinations. The awareness of patients during therapy is shifted from respiration to voice and articulation. Asthma impairs perceptual voice quality, maximum phonation time, frequency and amplitude perturbation parameters [24]. Thus, it is not surprising that patients receiving ATS appreciate the voice-work as an important part of ATS.

Keeping in mind that we performed a total of 539 individual therapy sessions in the trial, side effects occurred only rarely. The physical symptoms seen, e.g. coughing, may occur in all voice therapies and could be dealt with accordingly. Adverse events during the waiting phase were not systematically recorded, and thus a comparison between the two phases was not possible.

Some patients’ asthma symptoms increased during spring due to the higher pollen count or during winter due to infections of the upper respiratory tract. We recruited patients during all seasons to distribute these influences evenly.

In planning this real-life study, we faced several methodological issues. Blinding of the patients was not possible, since ATS requires the active participation of the patients, which is a limitation of this study. Many patients were reluctant to be randomly assigned to a control group without receiving the test therapy, i.e. speech therapy. Thus, a parallel-group design with sufficient power was not feasible. Performing a crossover trial allowed all participants to receive therapy, though dropouts, trends and carryover effects would represent general difficulties in the analysis. In this trial, dropouts only occurred in the ATS phase, mostly due to reasons unrelated to the therapy (e.g. infection or a leg fracture, Fig. 1). Randomisation took place before consent to participate in the study was received, and some patients allocated to waiting in the first phase decided not to participate.

A trend effect was visible during the first phase in the waiting group in some outcomes (e.g. overall AQLQ score). Context and meaning effects (e.g. patients’ expectations and monitoring of peak flow and medication intake) may have contributed to this finding. However, the study design does not allow us to discern if this effect continued during the second phase. In the group performing ATS first, AQLQ scores did not drop significantly during the subsequent waiting phase (Fig. 2), suggesting there was a considerable training effect (carryover).

On average, the patients participating in the trial experienced a positive outcome in asthma-related quality of life and asthma control, which continued after the crossover to the waiting phase. However, this carryover effect hampered our statistical analysis of the trial and reduced the practical advantages of the crossover study design. Thus, compliance and trial-design conflicts should be considered carefully in future studies of ATS. Researchers may also consider a longer treatment period and more intense regular practice at home.

## Conclusions

Breathing exercises are the most commonly used CM method for patients with asthma. In this randomised controlled crossover trial, we were able to demonstrate that ATS significantly improves asthma control and quality of life in patients with poorly controlled asthma. The side effects of ATS were only minor and occurred rarely. Whether ATS also improves lung function remains to be shown. In future trials, a longer treatment period and more intense regular practice at home may be considered.

## Additional file

Additional file 1: CONSORT 2010 checklist of information to include when reporting a randomised trial. (PDF 57 kb)

## Abbreviations

ACT: Asthma Control Test
AEM: Anthroposophically extended medicine
AQLQ: Asthma Quality of Life Questionnaire
ATS: Anthroposophic therapeutic speech
CM: Complementary medicine
CONSORT: Consolidated Standards of Reporting Trials
FEV1: Forced expiratory volume in 1 s
FVC: Forced expiratory vital capacity
IQR: Interquartile range
SD: Standard deviation

## Footnotes

1 Details of the exercises are available from the authors.

## References

[1] Global Initiative for Asthma (GINA). Global strategy for asthma management and prevention. 2016; https://ginasthma.org/wp-content/uploads/2016/04/wms-GINA-2016-main-report-final.pdf. Accessed 22 June 2018.

[2] British Thoracic Society, Scottish Intercollegiate Guidelines Network. British guideline on the management of asthma. 2014; https://www.brit-thoracic.org.uk/document-library/clinical-information/asthma/btssign-asthma-guideline-2014/. Accessed 22 June 2018.

[3] O’Connor E, Patnode CD, Burda BU, Buckley DI, Whitlock EP (2012). Breathing exercises and/or retraining techniques in the treatment of asthma: comparative effectiveness.

[4] Freitas DA, Holloway EA, Bruno SS, Chaves GSS, Fregonezi GAF, Mendonça KMPP (2013). Breathing exercises for adults with asthma. Cochrane Database Syst Rev.

[5] Grammatopoulou EP, Skordilis EK, Stavrou N, Myrianthefs P, Karteroliotis K, Baltopoulos G, Koutsouki D (2011). The effect of physiotherapy-based breathing retraining on asthma control. J Asthma.

[6] Burgess J, Ekanayake B, Lowe A, Dunt D, Thien F, Dharmage SC (2011). Systematic review of the effectiveness of breathing retraining in asthma management. Expert Rev Respir Med.

[7] Silva IS, Fregonezi GAF, Dias FAL, Ribeiro CTD, Guerra RO, Ferreira GMH (2013). Inspiratory muscle training for asthma. Cochrane Database Syst Rev.

[8] Slader CA, Reddel HK, Jenkins CR, Armour CL, Bosnic-Anticevich SZ (2006). Complementary and alternative medicine use in asthma: who is using what?. Respirology.

[9] Chen W, FitzGerald JM, Rousseau R, Lynd LD, Tan WC, Sadatsafavi M (2013). Complementary and alternative asthma treatments and their association with asthma control: a population-based study. BMJ Open.

[10] Kienle GS, Albonico HU, Baars E, Hamre HJ, Zimmermann P, Kiene H (2013). Anthroposophic medicine: an integrative medical system originating in Europe. Glob Adv Health Med.

[11] Hamre HJ, Witt CM, Kienle GS, Glockmann A, Ziegler R, Willich SN, Kiene H (2009). Long-term outcomes of anthroposophic therapy for chronic low back pain: a two-year follow-up analysis. J Pain Res.

[12] Hamre HJ, Witt CM, Kienle GS, Schnürer C, Glockmann A, Ziegler R, Willich SN, Kiene H (2009). Anthroposophic therapy for asthma: a two-year prospective cohort study in routine outpatient settings. J Asthma Allergy.

[13] Denjean-von Stryk B, von Bonin D (2003). Anthroposophical therapeutic speech.

[14] Cysarz D, von Bonin D, Lackner H, Heusser P, Moser M, Bettermann H (2004). Oscillations of heart rate and respiration synchronize during poetry recitation. Am J Physiol Heart Circ Physiol.

[15] Reddel HK, Taylor DR, Bateman ED, Boulet LP, Boushey HA, Busse WW, Casale TB, Chanez P, Enright PL, Gibson PG, de Jongste JC, Kerstjens HA, Lazarus SC, Levy ML, O’Byrne PM, Partridge MR, Pavord ID, Sears MR, Sterk PJ, Stoloff SW, Sullivan SD, Szefler SJ, Thomas MD, Wenzel SE (2009). American Thoracic Society/European respiratory task force on asthma control and exacerbations. An official American Thoracic Society/European Respiratory Society statement: asthma control and exacerbations: standardizing endpoints for clinical asthma trials and clinical practice. Am J Crit Care Med.

[16] Juniper EF, Guyatt GH, Ferrie PJ, Griffith LE (1993). Measuring quality of life in asthma. Am Rev Respir Dis.

[17] Nathan RA, Sorkness CA, Kosinski M, Schatz M, Li JT, Marcus P, Murray JJ, Pendergraft TB (2004). Development of the asthma control test: a survey for assessing asthma control. J Allergy Clin Immunol.

[18] Senn S (2002). Cross-over trials in clinical research.

[19] Juniper EF, Guyatt GH, Willan A, Griffith LE (1994). Determining a minimal important change in a disease-specific quality of life questionnaire. J Clin Epidemiol.

[20] Thomas M, Kay S, Pike J, Williams A, Rosenzweig JRC, Hillyer EV, Price D (2009). The asthma control test (ACT) as a predictor of GINA guideline-defined asthma control: analysis of a multinational cross-sectional survey. Prim Care Respir J.

[21] Schatz M, Kosinski M, Yarlas AS, Hanlon J, Watson ME, Jhingran P (2009). The minimally important difference of the asthma control test. J Allergy Clin Immunol.

[22] Axelsson M, Ekerljung L, Lundbäck B (2015). The significance of asthma follow-up consultations for adherence to asthma medication, asthma medication beliefs, and asthma control. Nurs Res Pract.

[23] Carranza Rosenzweig JR, Edwards L, Lincourt W, Dorinsky P, ZuWallack RL (2004). The relationship between health-related quality of life, lung function and daily symptoms in patients with persistent asthma. Respir Med.

[24] Dogan M, Eryuksel E, Kocak I, Celikel T, Sehitoglu MA (2007). Subjective and objective evaluation of voice quality in patients with asthma. J Voice.

